# Effect of iron‐fortified infant cereal on nutritional status of infants in Ghana

**DOI:** 10.1002/fsn3.2669

**Published:** 2021-11-26

**Authors:** Obed Akwaa Harrison, Nicholas P. Hays, Richard S. Ansong, Dominic Datoghe, Frederick Vuvor, Matilda Steiner‐Asiedu

**Affiliations:** ^1^ Department of Nutrition and Food Science University of Ghana Legon‐Accra Ghana; ^2^ Nestlé Product Technology Center‐ Nutrition Vevey Switzerland

**Keywords:** Africa, anemia, complementary food, fortified infant cereals, infant cereals, infants, iron fortification, iron status, nutritional status

## Abstract

Iron deficiency anemia is prevalent among infants in Ghana. This study evaluated the effect of micronutrient‐fortified infant cereal on the nutritional status of infants in the La Nkwantanang Municipality of the Greater Accra Region of Ghana, located in western Africa. In this double‐blind, controlled trial, infants aged 6–18 months were cluster‐randomized to receive either micronutrient‐fortified infant cereal containing 3.75 mg iron as ferrous fumarate/50 g cereal (INT; *n* = 107) or the same cereal without iron (CTL; *n* = 101) to complement other foods and breast milk. The intervention phase lasted six months followed by a two‐month post–intervention phase (with no further study product feeding). Hemoglobin and anthropometry were assessed every 2 months for the 8‐month study period. After the 6‐month intervention phase, adjusted mean ± standard error change in hemoglobin from baseline in INT and CTL was 1.97 ± 0.19 and 1.16 ± 0.21 g/dl, respectively (*p* < .01 for each); the increase in hemoglobin was significantly larger in INT versus CTL (increase 0.68 ± 0.30 g/dl; *p* = .02). Prevalence of anemia declined to a significantly greater extent in INT (84.1% to 42.8%) compared to CTL (89.1% to 62.8%; *p* = .006). There was no significant difference between groups in weight gain (*p* = .41) or height gain (*p* = .21) over the study period. In infants aged 6–18 months, micronutrient‐fortified infant cereal consumed for 6 months promoted greater reductions in iron‐deficiency anemia, which is a significant public health concern not only in Ghana but also in many developing countries globally.

## Graphical abstract

In Ghana, >65% of children aged 0–5 years are affected by anemia. Micronutrient‐fortified infant cereal providing 3.75 mg of iron fumarate per 50 g for 6 months improved hemoglobin and iron status and decreased the prevalence of anemia among infants and young children aged 6 to 18 months.

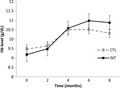

## 1 INTRODUCTION

Adequate nutrition is essential for proper growth and development during the first two years of life (World Health Organization, 2009). Globally, nearly 200 million children are chronically malnourished and suffer from serious, often irreversible, physical and cognitive impairments (World Food Program USA, 2015). In the country of Ghana, on the western coast of Africa, the Multiple Indicator Cluster Survey found that 23% of children under the age of five years were moderately or severely stunted, 6% wasted, and 13% underweight (Ghana Statistical Service, 2012). The pattern is slightly better in the administrative region including the nation's capital (the Greater Accra Region of Ghana) with 10.4% of children under 5 years considered to be stunted, 3.7% wasted, and 8.7% underweight (Ghana Statistical Service (GSS), Ghana Health Service (GHS), and ICF International, 2015). Despite improvements in the country's economy and available interventions to lower the prevalence and burden of poor nutritional status, malnutrition remains a major challenge in Ghana.

Malnutrition is a leading cause of anemia, and iron deficiency is responsible for approximately half of all cases of anemia (Lemoine & Tounian, 2020; Mantadakis et al., 2020). Iron deficiency and anemia can impair cognitive development, stunt growth, and increase morbidity (Lozoff, 2007). While the global prevalence of anemia in children under 5 years decreased from 47% in 1993–2005 (World Health Organization, 2008) to 43% in 2011 (World Health Organization, 2015), further reduction, unfortunately, has not been achieved, with a global prevalence of 42% observed in 2016 (World Health Organization, 2016a). In Ghana, the prevalence of anemia is higher than the global figures with a prevalence of 67% among children under 5 years in 2016 (The World Bank, 2021).

This high prevalence of anemia can be partly attributed to poor maternal nutrition before and during pregnancy and to poor nutrition during the first 2 years of the infants’ lives. WHO guidelines indicate that daily iron supplementation or fortification is recommended for the prevention of iron deficiency and anemia in infants and toddlers aged 6 to 23 months living in anemia‐prevalent regions (World Health Organization, 2016a, 2016b). A recent double‐blind, cluster‐randomized, controlled study evaluating the impact of iron‐fortified infant cereal among anemic children aged 18 to 59 months in Cameroon demonstrated significant reductions in the prevalence of anemia, iron deficiency, and iron deficiency anemia and higher weight‐for‐age z‐scores among infants who received the iron‐fortified cereal compared with controls (Ekoe et al., 2020).

Given the impact of iron fortification on this population of toddlers and considering WHO’s recommendation to initiate daily iron fortification at age 6 months in at‐risk regions (World Health Organization, 2016a, 2016b), we conducted a similar trial in the La Nkwantanang district of the Greater Accra region of Ghana. This cluster‐randomized controlled trial was designed to evaluate the efficacy of an infant cereal containing iron and other micronutrients on improving the nutritional status, particularly measured through hemoglobin levels, of infants and young children aged 6 to 18 months.

### 1.1 Key messages

- In Ghana, >65% of children aged 0–5 years are affected by anemia, much of which due to iron deficiency.
- The consumption of micronutrient‐fortified infant cereal providing 3.75 mg of iron fumarate per 50 g for 6 months improved hemoglobin concentrations and decreased the prevalence of anemia among infants and young children aged 6 to 18 months.

## 2 METHODS

### 2.1 Sampling method

**FIGURE 1:**
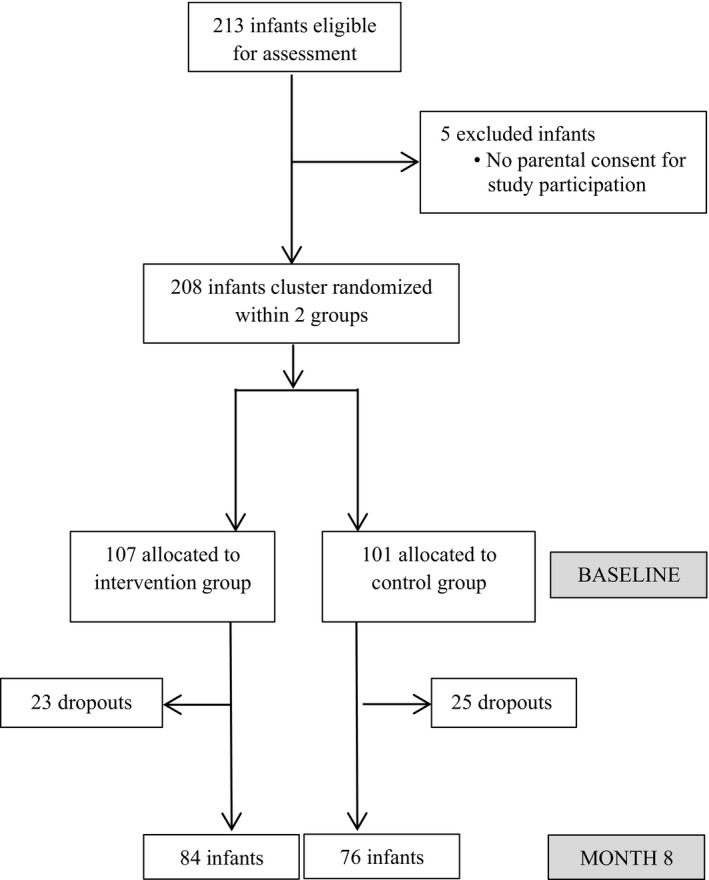
Flow of participant inclusion into the trial

This double‐blind, cluster‐randomized, controlled trial was conducted in the La Nkwantanang Municipal district of the Greater Accra region of Ghana from May 2016 to June 2017. Of the 15 peri‐urban communities in this district, one‐third (5/15) were randomly selected for this trial (Ayi Mensah, Danfa, Kweiman, Adoteiman, and Otinibi; for locations, see Figure S1). After reviewing the results of a census conducted in these five communities, the total area was demarcated into two regional clusters. These two clusters were randomly assigned to receive either the test cereal or the control cereal. This approach to randomization was used to prevent treatment contamination that can occur if randomized participants share study food, which is less likely if the two groups reside in geographically distinct regions. Study personnel recruited infants for this trial through house‐to‐house visits in these five communities. Infants and young children aged 6–18 months and in apparent good health were eligible for enrollment. Infants who were sick, on medication due to a recent ailment, or allergic to wheat‐based foods were excluded from the study. Infants were assessed for edema prior to recruitment and during every assessment time point throughout the study. There were 213 infants eligible for inclusion, but informed consent could only be obtained for 208. These two groups were randomly assigned to the intervention (INT, *n* = 107) or control (CTL, *n* = 101) (Figure 1). Infants in the CTL group were provided with micronutrient‐fortified infant cereal without iron to complement other family foods and breast milk, and those in the INT group were provided with identical micronutrient‐fortified infant cereal containing iron. Table S1 shows the nutrient profile of both cereals. The iron‐fortified infant cereal provided 3.75 mg iron (as ferrous fumarate) per 50 g. Infants aged 6 to 8 months received 50 g/day, which provided 210 kcal of energy; infants 9 to 11 months of age received 75 g/day, which provided 320 kcal of energy; and infants 12 to 18 months of age received 100 g/day, which provided 420 kcal of energy (Dewey & Brown, 2003). All investigators and caregivers were blinded to the cereal assignment.

### 2.2 Sample size calculation

To obtain an alpha level (α) of 0.05 or 5% and statistical power (1‐β) of 90%, a mean hemoglobin level of 9.4 g/dl, a standard deviation of 1.6 g/dl, and an increase of 1.0 g/dl in the iron‐fortified group between baseline and study completion at 6 months, the minimum sample size was calculated to be 54 in each group, that is, 108 subjects for the entire study (Charan & Biswas, 2013). The size was increased to 100 per group to account for a potentially high attrition rate, which can occur in clinical trials that involve blood draws (Adjei & Enuameh, 2015).

### 2.3 Procedure and outcomes

The study duration was eight months including the six‐month nutritional intervention phase and then a post–intervention evaluation phase (with no study product feeding) lasting two months after the intervention phase.

During the 6‐month intervention period, the infants received weekly rations of the cereal brought to their home by study personnel, which also provided frequent opportunities for interaction and gathering feedback from the mothers/caregivers. In addition, weekly follow‐up calls were conducted by study personnel to monitor the feeding process. Sachets of cereal were collected on a weekly basis and weighed to measure the amount of cereal consumed by infants.

Sociodemographic characteristics of infants and mothers/caregivers, including age, household size, level of education, and occupation were obtained using a semi‐structured, pretested questionnaire. Information on infants’ usual dietary intake, including the diversity and frequency of various foods, was assessed. The dietary diversity part of the questionnaire inquired about food items from all food groups and other vitamin A‐rich cereals or complementary foods that were consumed in the last 24 hr before the interview. Dietary diversity score and feeding frequency were estimated from these data. Dietary status was evaluated by assessing the consumption of the following nine food groups: a) grains, roots, and tubers, b) legumes and nuts, c) dairy products, d) flesh foods, e) eggs, f) vitamin A‐rich fruits and vegetables, g) other fruits, h) other vegetables, and i) fats and oils. Infants were classified as having low, moderate, or highly diversified diets based on the consumption of items from <4, 4, or >4 of these groups, respectively.

Anthropometric data were collected at baseline and study visits occurring 2, 4, 6, and 8 months after study initiation. Data collection for weight, recumbent length, and height was performed by qualified nutritionists who were trained in conducting anthropometric measurements and supervised by the principal investigator and/or co‐investigators. These data were used to determine *z*‐scores for weight‐for‐age, height‐for‐age, and weight‐for‐height at each point of data collection. The infants were weighed using a Salter hanging scale, which was properly calibrated prior to each measurement, and recumbent length was recorded with an infantometer. Mid‐upper arm circumference (MUAC) was measured with non‐stretchable MUAC tapes. All measurements were repeated twice, and the mean values were recorded.

Hemoglobin levels were measured via a drop of capillary blood taken from the infant's fingertip or heel using the portable HemoCue Haemoglobinometer (HemoCue AB, Ängelholm, Sweden) at baseline and 2, 4, 6, and 8 months of study. Worm infestation and confirmatory microscopic malaria tests were done at the Immunology Department of Noguchi Memorial Research Institute to identify whether these conditions were present in the infants and to monitor their effect on hemoglobin levels. Malaria status was assessed using the rapid diagnostic test followed by the microscopy technique, which was applied as a confirmatory procedure (Moody, 2002). Clinical signs were examined in the following areas: gum, teeth, tongue, mouth, lips, hair, skin, nails, and eyes. Observations were compared with standard charts by trained study personnel to identify whether there were any abnormal clinical signs in the infants.

### 2.4 Ethics

The Institutional Review Board of the Noguchi Memorial Institute for Medical Research, University of Ghana, Legon, approved this study. Before data collection, chiefs and community elders were informed and their permission was obtained after a thorough explanation of the study procedures. Written informed consent was also obtained from the parents/caregivers of all the recruited infants after a thorough explanation of the objectives, nature, and risks of the study. The trial was registered retrospectively with the Pan African Clinical Trial Registry registration number: PACTR201906885776793.

### 2.5 Statistical analysis

The primary endpoint of the study was iron deficiency anemia. Consistent with the WHO anemia definition for children under 5, the presence of anemia was defined as a hemoglobin level of <11 g/dl (McLean et al., 2009). Nutritional status indicators such as weight and height were secondary endpoints.

Data were analyzed using SPSS (version 21.0; SPSS Inc., Chicago, IBM) and WHO Anthro software (version 3.2.2; WHO, Geneva) to evaluate the full analysis dataset, which included all enrolled subjects who received at least one serving of the study cereal and who had post‐enrollment data. The WHO Anthro software was used to calculate the weight‐for‐age, height‐for‐age, and weight‐for‐height *z*‐scores of the infants to determine their nutritional status as indicated by the presence of stunting, wasting, and underweight in comparison with the WHO standard reference population. Descriptive statistics, including frequencies and percentages for categorical variables and mean and standard deviation for continuous variables, were utilized. Baseline characteristics including demographics and anthropometrics were compared between the INT and CTL groups using chi‐squared tests (for frequencies) and *t*‐tests (for continuous variables). Mean change in hemoglobin values from baseline to both 6 months and 8 months was computed within each group overall and by sex and then compared within groups using paired *t*‐tests. Analysis of covariance (ANCOVA) was used to compute adjusted mean values for change in nutritional status indicators (including hemoglobin, anthropometric *z*‐scores, weight, and height) from baseline to 6 months in the INT and CTL groups after adjusting for malaria status, worm infestation, dietary diversity score, and mothers’ education. Chi‐squared tests (for frequencies) and *t*‐tests (for continuous variables) were used to determine whether changes from baseline to the end of the study were statistically significant between the two groups. The threshold of significance for the various tests was set at an alpha of 0.05.

## 3 RESULTS

Of the 208 infants included in the study, 107 were assigned to the iron‐fortified (INT) group and 101 were assigned to the control (CTL) group. Of the infants, 160 were evaluated at the end of the intervention; the drop‐out rate was 21% (23/107) and 25% (25/101) in the INT and CTL groups, respectively (Figure 1).

The demographic characteristics and nutritional status of the enrolled infants in addition to maternal and household characteristics at baseline are presented in Table 1. Infants in both groups were aged between 6 and 18 months, and there were no significant differences at baseline between the INT and CTL groups in terms of sex, height, weight, mid‐upper arm circumference, and maternal socioeconomic status. Mean Hb at baseline was 9.20 ± 1.61 g/dl in the INT group and 9.46 ± 1.49 g/dl in the CTL group.

**TABLE 1:** Baseline demographic and maternal and household characteristics of infants receiving micronutrient‐fortified infant cereal with (INT) or without (CTL) supplemental iron, shown as *n* (%) or mean ± *SD*

Characteristics	CTL (*n* = 101)	INT (*n* = 107)	*p*‐value
Age (months; mean ± *SD*)	12 ± 4	13 ± 4	.26
6–8	28 (28.0%)	15 (14.6%)	.05
9–11	24 (24.0%)	23 (22.3%)	−
12–15	22 (22.0%)	37 (35.9%)	−
16–18	26 (26.0%)	28 (27.2%)	−
Sex, *n* (%)			
Boy	58 (57.4%)	48 (44.9%)	.07
Girl	43 (42.6%)	57 (52.3%)	−
Weight, kg			
Boys	9.01 ± 1.50	8.65 ± 1.61	.23
Girls	9.20 ± 1.39	8.63 ± 1.31	.05
Height, cm			
Boys	74.55 ± 5.55	73.09 ± 5.37	.17
Girls	75.66 ± 4.51	73.82 ± 5.96	.09
MUAC, cm	14.66 ± 1.32	14.42 ± 1.26	.19
Stunted, *n* (%)	5 (5.0%)	11 (10.5%)	.14
Underweight, *n* (%)	5 (5.0%)	13 (12.4%)	.06
Wasted, *n* (%)	4 (4.0%)	13 (12.1%)	.09
Overweight, *n* (%)	3 (3.0%)	4 (3.7%)	.09
Prevalence of anemia, *n* (%)	85 (84.1)	95 (89.1)	.33
Maternal occupation			
Not employed	16 (15.8%)	17 (15.9%)	.99
Part‐time labor	5 (5.0%)	4 (3.7%)	.67
Full‐time salary	3 (3.0%)	11 (10.3%)	.05
Selling goods	47 (46.5%)	47 (43.9%)	.59
Student	2 (2.0%)	0 (0.0%)	.14
Artisan	30 (29.7%)	28 (26.2%)	.57
Maternal educational status			
None	8 (8.0%)	11 (10.3%)	.05
Primary	24 (24.0%)	22 (20.6%)	−
Middle	15 (15.0%)	21 (19.6%)	−
Secondary/Vocational	51 (51.0%)	41 (38.3%)	−
Tertiary	2 (2.0%)	12 (11.2%)	−

Abbreviations: *SD* = Standard deviation; MUAC = mid‐upper arm circumference; nutritional status (stunting, underweight, wasting, overweight) was determined based on WHO *z*‐scores.

In the INT and CTL groups, the mean change in hemoglobin levels was 1.90 ± 1.81 g/dl (*p* < .01) and 1.22 ± 1.83 g/dl (*p* < .01), respectively, at the end of the 6‐month intervention phase (Table 2). The difference between the two groups in the mean change was significantly larger among infants receiving the iron‐fortified cereal (0.68 ± 0.30 g/dl; *p* = .02). A similar trend was observed at the end of the 8‐month study period, which included the post–intervention phase; the magnitude of the increase in hemoglobin levels was again higher in the INT group (1.68 ± 1.72 g/dl; *p* < .01) than in the CTL group (0.89 ± 1.87 g/dl; *p* < .01) (Table 2).

**TABLE 2:** Mean changes in hemoglobin concentrations (g/dL) from baseline to month 6 and month 8 among 208 infants receiving micronutrient‐fortified infant cereal with (INT) or without (CTL) supplemental iron

	Baseline to 6 months (intervention period)				Baseline to 8 months (intervention period + post–intervention period)			
	CTL		INT		CTL		INT	
	Change in hemoglobin	*p*‐value	Change in hemoglobin	*p*‐value	Change in hemoglobin	*p*‐value	Change in hemoglobin	*p*‐value
Males	0.92 ± 1.85	< .01	2.11 ± 1.92	< .01	0.94 ± 2.04	< .01	2.12 ± 1.83	< .01
Females	1.71 ± 1.73	< .01	1.73 ± 1.70	< .01	0.81 ± 1.56	.01	1.30 ± 1.54	< .01
All	1.22 ± 1.83	< .01	1.90 ± 1.81	< .01	0.89 ± 1.87	< .01	1.68 ± 1.72	< .01

Note

Changes are reported as mean ± *SD*.

**FIGURE 2:**
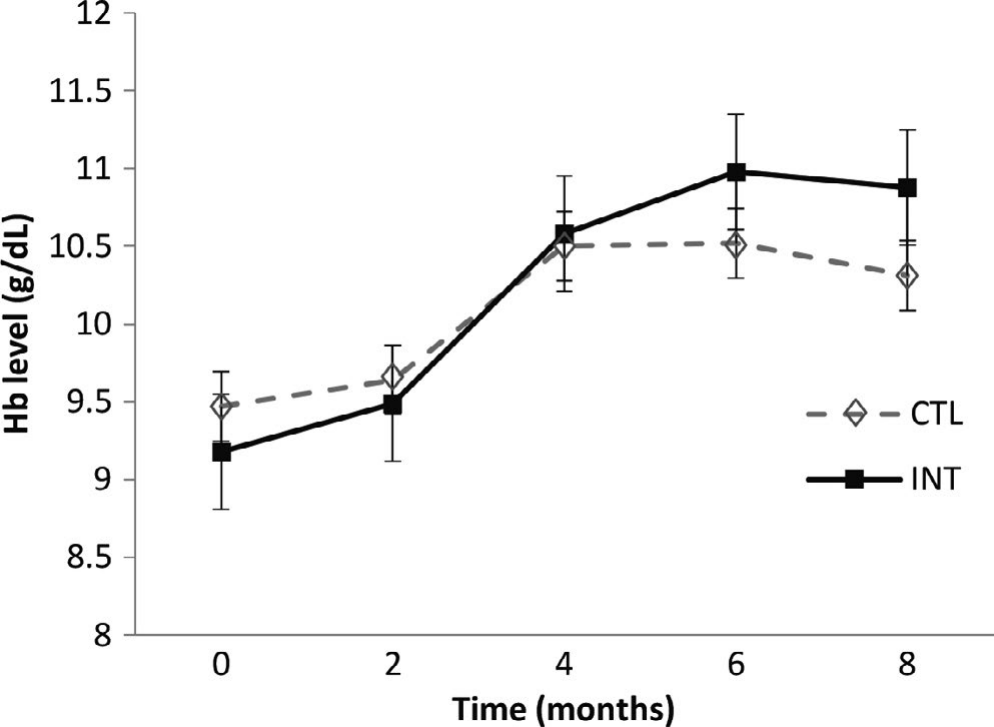
Hemoglobin concentrations from baseline to end of the study period (month 8) among 208 infants receiving micronutrient‐fortified infant cereal with (INT) or without (CTL) supplemental iron

The adjusted mean change in hemoglobin levels was significantly different between the INT and CTL groups from baseline to month 6 (1.97 ± 0.19 versus 1.16 ± 0.21 g/dl; *p* < .01; Table 3). Although hemoglobin concentration slightly decreased between the 6th and 8th month, the INT group still had higher hemoglobin levels than the CTL group at 8 months (10.88 ± 1.18 g/dl and 10.35 ± 1.24 g/dl, respectively; *p* < .01) (Figure 2). Lastly, the prevalence of anemia decreased to a significantly greater extent in the group receiving the iron‐fortified cereal; anemia prevalence declined from 84.1% to 42.8% in the INT group and from 89.1% to 62.8% in the CTL group from baseline to the end of the study period (8 months; *p* = .006).

**TABLE 3:** Changes from baseline to month 6 in anthropometric parameters among 208 infants receiving micronutrient‐fortified infant cereal with (INT) or without (CTL) supplemental iron

Indicators	Unadjusted change from baseline			Adjusted change from baseline		
	CTL (Mean ± SE)	INT (Mean ± SE)	*p*‐value	CTL (Mean ± SE)	INT (Mean ± SE)	*p*‐value
Hemoglobin concentration, g/dL	1.22 ± 0.22	1.90 ± 0.20	.02	1.16 ± 0.21	1.97 ± 0.19	< .01
Weight‐for‐age *z*‐score	0.87 ± 0.19	1.06 ± 0.19	.48	0.99 ± 0.19	1.13 ± 0.20	.61
Height‐for‐age *z*‐score	2.28 ± 0.26	2.30 ± 0.25	.95	2.47 ± 0.27	2.54 ± 0.27	.84
Weight‐for‐height *z*‐score	−0.21 ± 0.18	0.11 ± 0.17	.21	−0.18 ± 0.19	0.06 ± 0.19	.38
Weight, kg	0.87 ± 0.23	1.20 ± 0.22	.30	1.03 ± 0.24	1.31 ± 0.24	.41
Height, cm	6.11 ± 0.27	5.55 ± 0.26	.14	6.24 ± 0.28	5.62 ± 0.27	.21

Note

Adjusted means derived from ANCOVA models controlling for malaria status, worm infestation, dietary diversity score, and mothers’ education.

The INT and CTL groups were comparable in weight, height, and mid‐upper arm circumference at baseline (Figure S2a‐c). The prevalence of stunting, underweight, wasting, and overweight was also similar in both groups at baseline. There was a gradual increase in mid‐upper arm circumference, height, and weight from baseline to month 6 in both groups, but the withdrawal of feeding in the post–intervention period (months 7 and 8) resulted in a slight decrease in mid‐upper arm circumference and weight in the INT group but not in the CTL group (Figure [Link], [Link], [Link]a‐c).

Changes from baseline to month 6 in anthropometric assessments, including *z*‐scores for weight‐for‐age, height‐for‐age, and weight‐for‐height, are presented in Table 3, after adjusting for worm infestation, malaria status, dietary diversity score, and mother's education. There were no significant differences between the INT and CTL groups for any anthropometric measure over time (*p* > .05).

At baseline, both the INT and CTL groups exhibited similar dietary diversity, except in the consumption of grains, roots, and tubers (92.5% versus 83.2%, respectively; *p* = .04); vitamin A‐rich fruits and vegetables (26.4% versus 9.9%, respectively; *p* < .04); and other fruits (31.1% versus 17.8%, respectively; *p* = .03) (data not shown). High dietary diversity (defined as food intake from 5–9 food groups) was found in 17.9% of the infants in the INT group compared with 7.9% in the CTL group at baseline (*p* ≤ .01) (Figure S3). Dietary diversity would be expected to improve over time as the infants transitioned to eating more complementary foods, and indeed, at 6 months, a greater proportion of infants exhibited high dietary diversity in both the INT and the CTL groups (42.2% and 25.3% respectively). The difference in the distribution of low, medium, and high dietary diversity at 6 months was no longer significantly different between the groups (*p* = .08). By month 8, however, when the intervention had been withdrawn, the infants in the INT group were exhibiting notably higher dietary diversity compared with those in the CTL group (65.1% versus 15.5%; *p* ≤ .01) (Figure S3).

## 4 DISCUSSION

This study found that daily consumption of cereal fortified with 3.75 mg of iron as ferrous fumarate for six months among infants and young children aged 6–18 months in the La Nkwantanang Municipal district of the Greater Accra region of Ghana improved hemoglobin levels and decreased the prevalence of anemia. Infants in the control group had slightly higher hemoglobin levels than those in the intervention group at baseline, but the increase in hemoglobin levels was significantly higher in the intervention group compared with the control group over the eight‐month study period.

The need for improvement in nutritional status, particularly related to anemia, is stark in this population. At baseline, the majority of the infants were anemic in both the intervention and control groups (89.1% and 84.1%, respectively), which is well above the reported 67% prevalence of anemia among children aged below 5 years in Ghana (The World Bank, 2021). Per WHO guidelines, 6 months is the recommended appropriate age for the introduction of complementary foods, as breast milk alone is insufficient for providing the required calories and nutrients (Sundararajan & Rabe, 2021). However, young children have a higher risk of undernutrition if the provided complementary foods are nutritionally inadequate; hence, fortified complementary foods or vitamin‐mineral supplements are recommended (World Health Organization, 2009). As the iron requirement is high in late infancy, iron fortification has emerged as a feasible and practical approach specifically for anemia risk reduction.

In this study, after a 6‐month intervention, we observed a 15% greater decline in the prevalence of anemia and a significantly larger increase in hemoglobin levels (0.68 g/dl) in the iron‐fortified cereal group compared with the control group. During the two months following the intervention, hemoglobin levels in the iron‐fortified group declined slightly but still remained higher than those in the control group. This indicates that consistent daily consumption of iron‐fortified cereal is important to maintain hemoglobin levels.

The results from this study regarding improvements in anemia are in line with previous findings from recent randomized clinical trials and systematic reviews. For example, in a study involving children aged 18 to 59 months from Cameroon, children who consumed an iron‐fortified infant cereal had significantly higher baseline‐adjusted mean hemoglobin levels after 6 months compared to controls (10.0 ± 1.8 versus 9.7 ± 1.4 g/dl; *p* = .023) and had a lower prevalence of iron‐deficiency anemia (14.6% versus 53.4%; *p* < .001) (Ekoe et al., 2020). Moreover, in a systematic review of 18 randomized trials using iron multi‐micronutrient‐fortified milk and cereal food in children aged 6 months to 5 years, which observed a mean increase of 0.87 g/dl in hemoglobin levels and a 50% reduction in the risk of anemia (Eichler et al., 2012). Similarly, a meta‐analysis of 54 randomized controlled trials found a significant mean increase of 0.42 g/dl in hemoglobin levels and a reduced risk of anemia in groups receiving fortified or biofortified foods compared with controls (Gera et al., 2012). The meta‐analysis was conducted in largely non‐malarial endemic regions, which are comparable to our study in which the prevalence of malaria was negligible.

There was no significant improvement observed in our study between the intervention and control groups in terms of weight or length gain, which could be due to the short duration of the follow‐up. Given that over 85% of the infants were anemic at baseline, the lack of observed improvement between the groups in growth metrics could also be due to the known association between iron deficiency and reduced appetite (Gao et al., 2015). Our results are in line with a systematic review of 25 randomized controlled trials, which evaluated the effect of iron supplementation on physical growth in children and did not find a statistically significant (*p* > .05) positive effect of iron supplementation on any anthropometric variable (weight‐for‐age *z*‐score, weight‐for‐height *z*‐score, height‐for‐age *z*‐score, and mid‐upper arm circumference) (Sachdev et al., 2006). In contrast, results from a recent study of children aged 18 to 59 months from Cameroon indicated that anemic children receiving iron‐fortified infant cereal had a very small but borderline significant higher weight gain (~50 g, *p* = .052) and significantly higher weight‐for‐age *z*‐score compared with the control group (−0.59 versus −1.03; *p* = .016), although no between‐group differences in height, height‐for‐age, and weight‐for‐height *z*‐scores were observed (Ekoe et al., 2020). However, it should be noted that the population in that study was mildly to moderately undernourished at baseline, with approximately 56% of children stunted in growth (height‐for‐age *z*‐scores < −2) and 25% underweight (weight‐for‐age *z*‐score < −2).

Results from the dietary data revealed a significant difference in dietary diversity at baseline, with a significantly higher percentage of children in the control group having a low dietary diversity score compared with children in the intervention group (83.2% versus 59.4%; *p* ≤ .01). Although this trend was expected to improve as the children transitioned into eating complementary foods, less than half of the children in both groups exhibited high dietary diversity at the end of the intervention. By the end of the study, 65.1% of participants in the intervention group exhibited high dietary diversity compared with 15.5% in the control group, suggesting that study participants experienced challenges with complementary feeding and that greater challenges were observed in the control group. Clearly then, the iron‐fortified or non‐iron‐fortified infant cereal has the potential to improve on the general nutritional status of children.

Strengths of this study include a large number of participants, a setting with high potential to receive benefit from the intervention, and weekly follow‐ups by study personnel to monitor the feeding process, as both dietary improvement and increased iron intake are necessary for improving nutritional status in infants. One potential limitation is that only two clusters were randomized, and the observed differences could be due to living conditions. However, demographics and other factors such as maternal education were generally similar between the two clusters, so it is unlikely that any differences are due to varying living conditions. Another possible limitation is that there was a slight imbalance between groups at baseline in age at enrollment and in sex, possibly resulting from differences in the communities within each cluster. Other potential limitations include the absence of assessments of other confounding factors such as vitamins A and B, and folate that affect hemoglobin levels, and the relatively short duration of the study which could have impacted the effectiveness of the intervention, particularly for the secondary outcomes of growth. In addition, the self‐reported 24‐hr dietary recall might have caused over‐ or under‐estimation of usual food intakes.

In conclusion, these results suggest that the consumption of micronutrient‐fortified infant cereal with 3.75 mg ferrous fumarate per 50 g as part of the daily diet improved hemoglobin and iron status and decreased the prevalence of anemia among infants aged 6–18 months. Given the impact of this intervention, the consumption of iron‐fortified cereals as part of the daily diet could help to improve micronutrient intake and nutritional status and lower the burden of iron‐deficiency anemia throughout Ghana and more broadly around the globe.

## CONFLICT OF INTEREST

NPH is an employee of Société des Produits Nestlé S.A. No other author has any conflict of interest to report.

## AUTHOR CONTRIBUTIONS

FV and MS‐A conceptualized the study; FV, OAH, and MS‐A were involved in study design and methodology; ; FV, OAH, DD, and MS‐A were involved in data collection; FV, RSA, and OAH conducted the data analysis; and FV, RSA, OAH, DD, NPH, and MS‐A wrote the manuscript. All authors read and approved the final manuscript.

## STUDY CONDUCT AND INFORMED CONSENT

This study was conducted in accordance with the informed consent guidelines stated in the Declaration of Helsinki and all applicable local laws and regulations. The study's protocols and procedures were ethically reviewed and approved by the Institutional Review Board of the Noguchi Memorial Institute for Medical Research, University of Ghana, Legon. Written informed consent was obtained from the parents/caregivers of all recruited infants.

## Supporting information

Fig S1

Fig S2a

Fig S2b

Fig S2c

Fig S3

Table S1

## Data Availability

The data that support the findings of this study are available from the corresponding author upon reasonable request.
