# Supplementary figures and images for: Effect of iron‐fortified infant cereal on nutritional status of infants in Ghana
Source: Food Sci Nutr. 2021 Nov 26;10(1):286–94. doi: 10.1002/fsn3.2669 (PMC8751428; doi:10.1002/fsn3.2669)

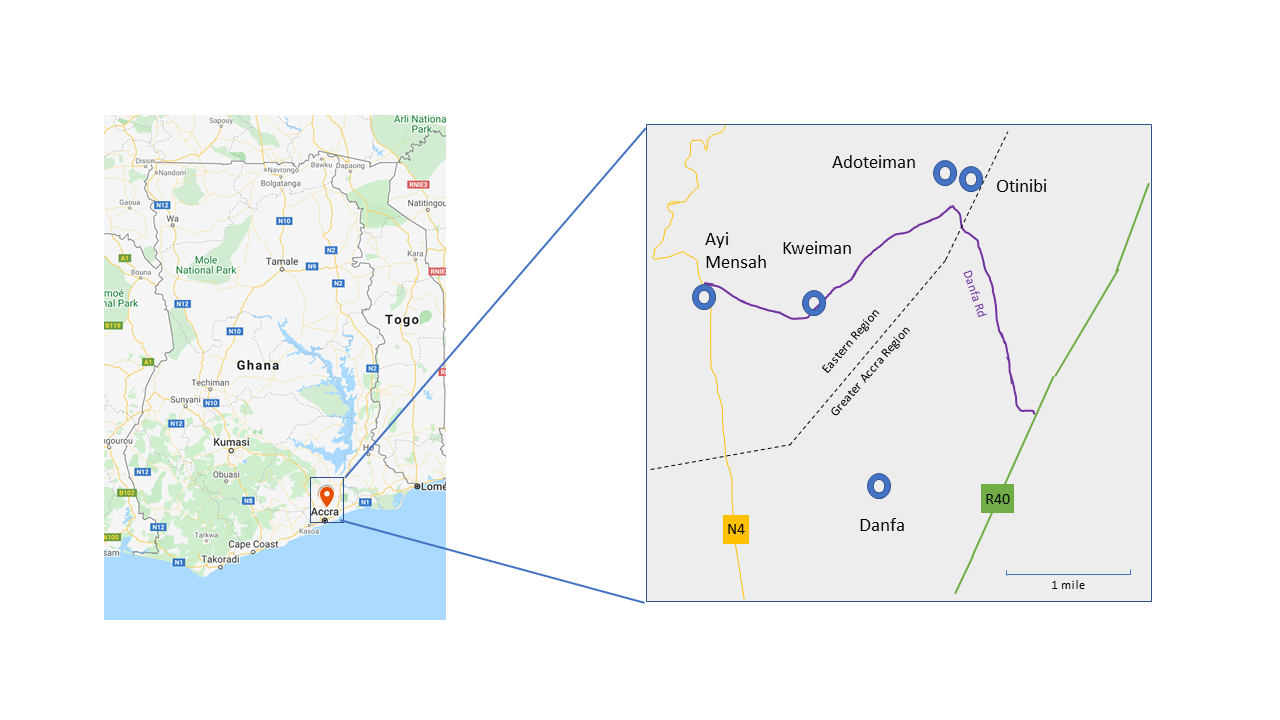

Supplement: Supplementary file 1 — Fig S1 [file FSN3-10-286-s006.tif]

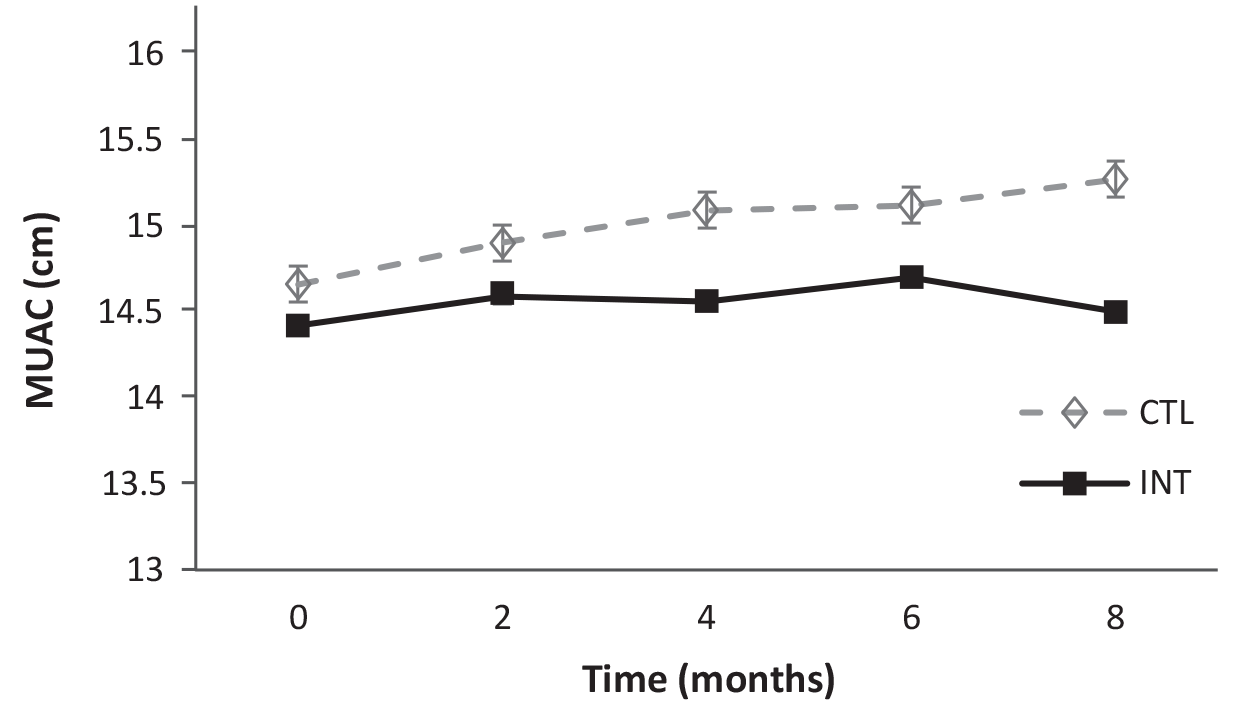

Supplement: Supplementary file 2 — Fig S2a [file FSN3-10-286-s003.tif]

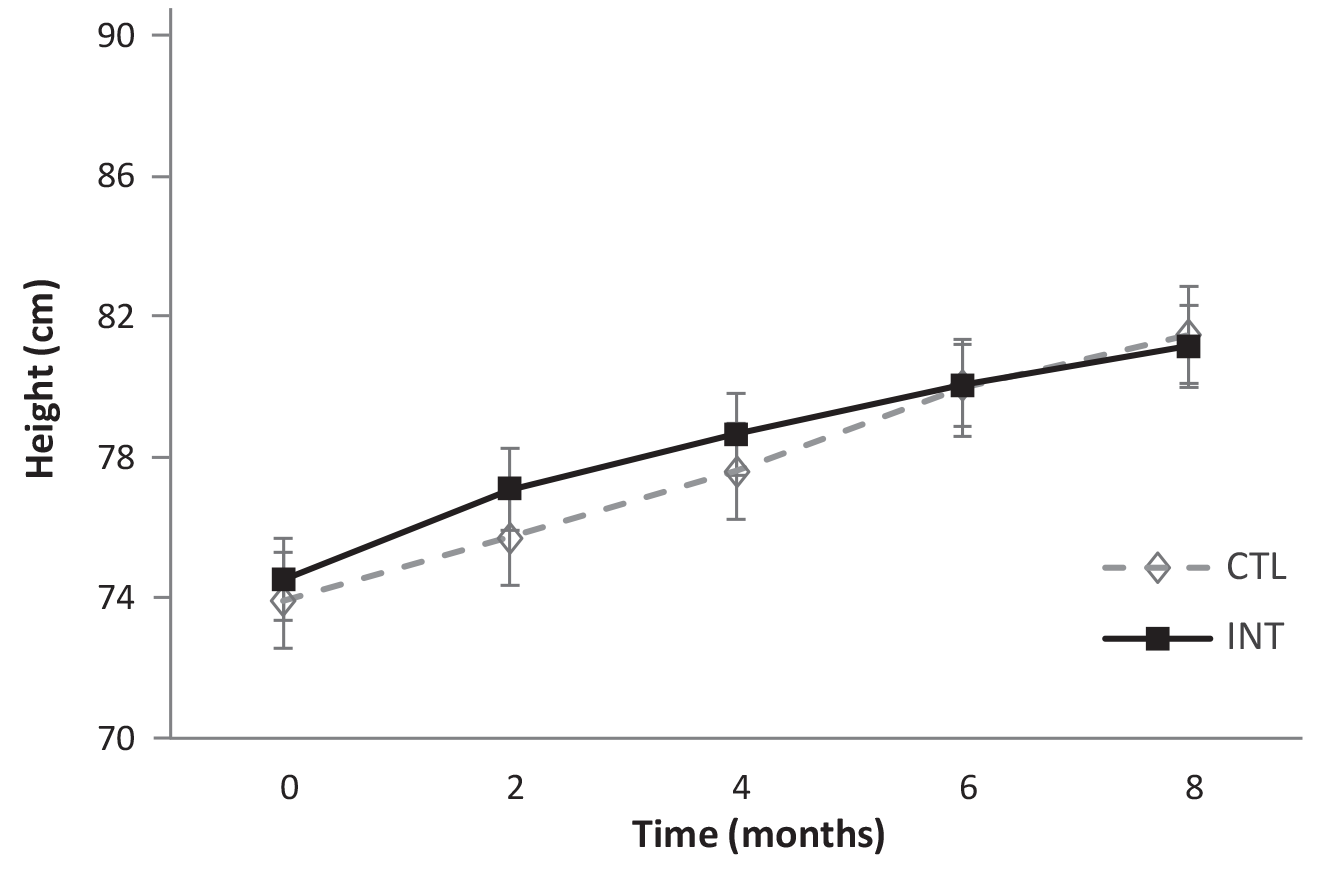

Supplement: Supplementary file 3 — Fig S2b [file FSN3-10-286-s002.tif]

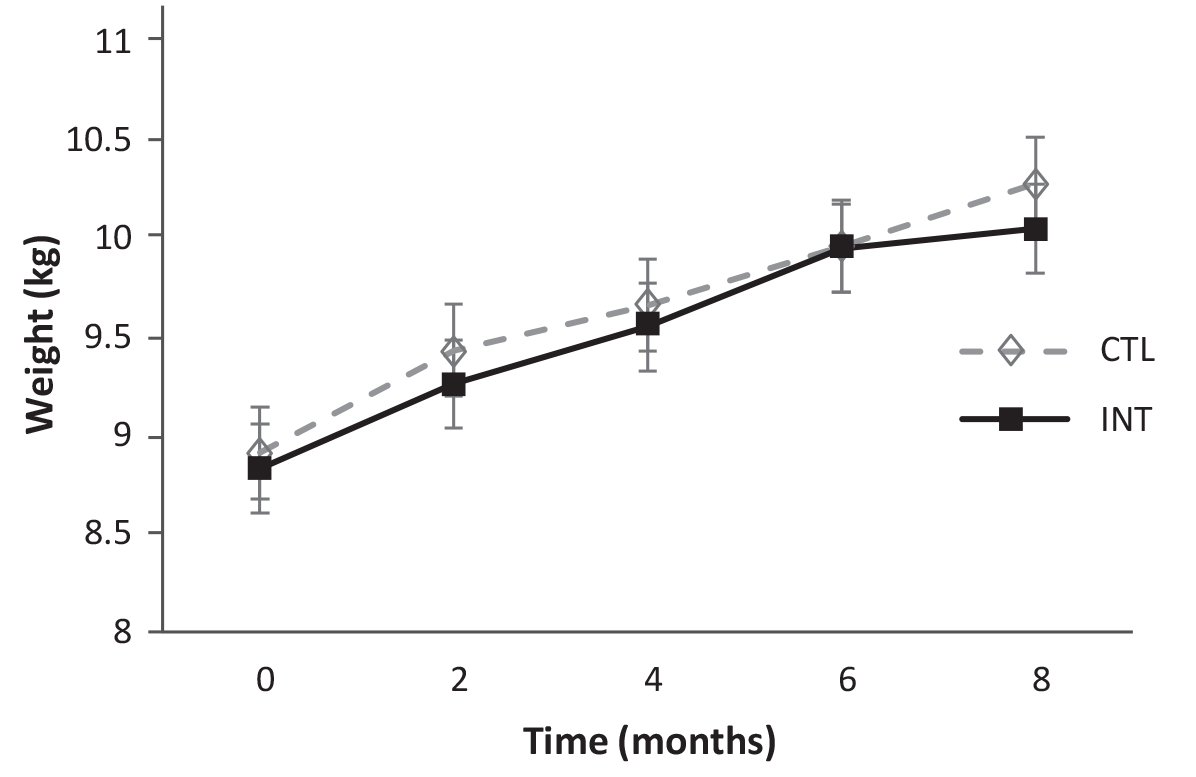

Supplement: Supplementary file 4 — Fig S2c [file FSN3-10-286-s001.tif]

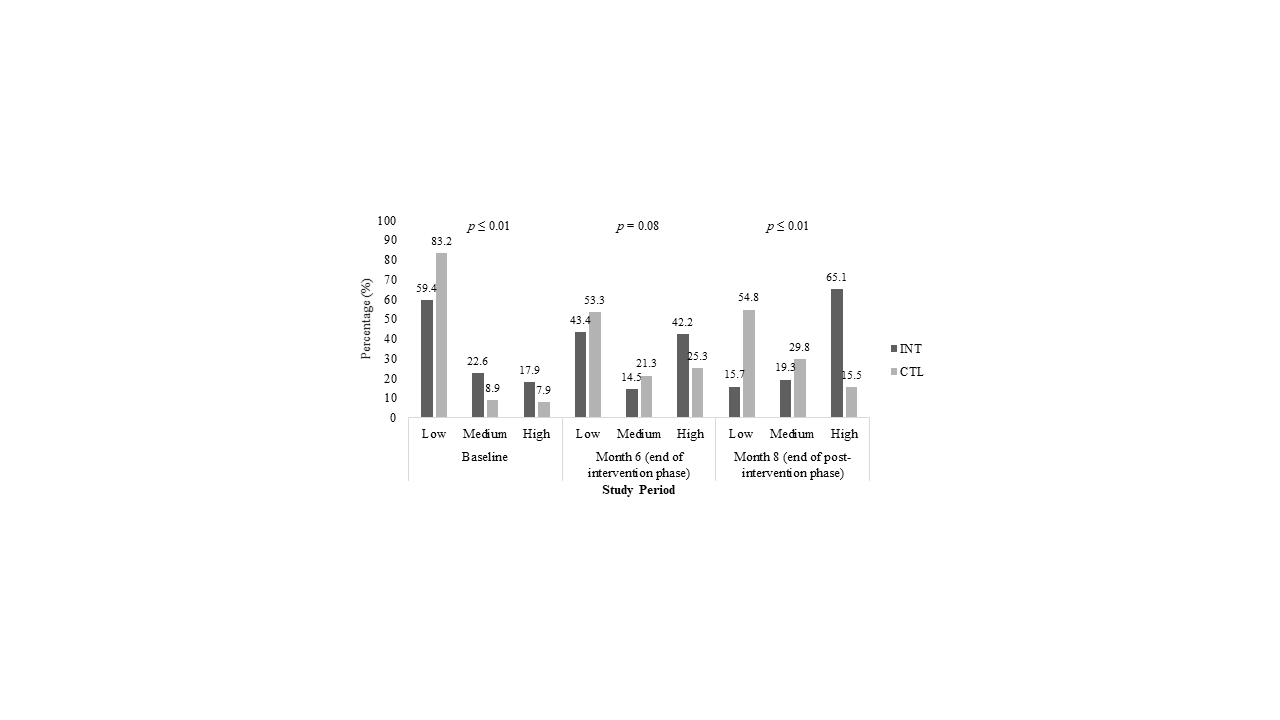

Supplement: Supplementary file 5 — Fig S3 [file FSN3-10-286-s004.tif]
