# Supplementary material for: Effect of iron‐fortified infant cereal on nutritional status of infants in Ghana
Source: Food Sci Nutr. 2021 Nov 26;10(1):286–94. doi: 10.1002/fsn3.2669 (PMC8751428; doi:10.1002/fsn3.2669)
Supplement: Supplementary file 6 — Table S1 [file FSN3-10-286-s005.docx]

**Table S1.** Infant cereal composition (nutrient content/50 g)

|  | **INT** | **CTL** |
| --- | --- | --- |
| Energy (kcal) | 210 | 210 |
| Fat (g) | 5 | 5 |
| Protein (g) | 7.25 | 7.25 |
| Carbohydrate (g) | 34 | 34 |
| Dietary fiber (g) | 1.4 | 1.4 |
| Sodium (mg) | 67.5 | 67.5 |
| Calcium (mg) | 225 | 225 |
| Iron (ferrous fumarate, mg) | 3.75 | 0 |
| Zinc (mg) | 2.5 | 2.5 |
| Vitamin A (IU) | 650 | 650 |
| Vitamin D (IU) | 90 | 90 |
| Vitamin C (mg) | 25 | 25 |
| Vitamin B1 (mg) | 0.3 | 0.3 |
